# Developmental Origins of Health and Disease: the relevance to developing nations

**DOI:** 10.1093/inthealth/ihy006

**Published:** 2018-03-08

**Authors:** Mirembe Mandy, Moffat Nyirenda

**Affiliations:** 1 Medical Research Council/Uganda Virus Research Institute Uganda Research Unit on AIDS, P Box 49, Entebbe, Uganda; 2 London School of Hygiene & Tropical Medicine, Keppel Street, London WC1E 7HT, UK

**Keywords:** Developmental origins of health and disease, Early-life, Low- and middle-income countries, Manutrition, Non-communicable diseases, Risk factors, Sub-saharan Africa

## Abstract

Low- and middle-income countries (LMICs), particularly those in sub-Saharan Africa, are experiencing rapid increases in the prevalence of non-communicable diseases (NCDs), which may not be fully explained by urbanization and associated traditional risk factors such as tobacco smoking, excessive alcohol consumption, poor diet or physical inactivity. In this commentary, we draw attention to the concept of Developmental Origins of Health and Disease (DOHaD), where environmental insults in early life can contribute to long-term risk of NCDs, the impact of which would be particularly important in LMICs where poverty, malnutrition, poor sanitation and infections are still prevalent.

## The concept

The ‘Developmental Origins of Health and Disease (DOHaD)’ hypothesis, a rather more recent term for the concept initially proposed and called ‘Fetal Origins of Adult Disease’ in the 1990s,^1^ postulates that exposure to certain environmental influences during critical periods of development and growth may have significant consequences on an individual’s short- and long-term health.^2^ In this concept, the developing fetus, if exposed to a hostile uterine environment (caused by insults such as poor nutrition, infections, chemicals, metabolite or hormonal perturbations),^3^ responds by developing adaptations (predictive adaptive responses—PARs), that not only foster its immediate viability, but also its survival if a similar environment is encountered later in life.^4,5^ Some examples of short-term adaptations the fetus may make in these scenarios include down-regulation of endocrine or metabolic function, and/or specific organ function to slow down its growth rate to match the nutrient supply in the deprived uterine environment.^6^ Long-term, subtle, irreversible changes in the development, structure and function of some tissues and vital organs (thymus, skeletal muscle, lungs, pancreas, kidney) may occur^7^ as a result of disruptions in gene expression, cell differentiation and proliferation. However, if the individual then grows up in an extra-uterine environment the reverse of that experienced in utero, the ‘mismatch’ and poorer fit, therefore, would predispose them to a higher risk of certain non-communicable diseases (NCDs).^3^ This risk is further exacerbated by excessive weight gain in postnatal/adult life, and by the aging process itself.^5,8^

## Manifestation of DOHaD

Much of the evidence underpinning DOHaD science has been obtained from animal models and observational human studies. It shows that the period from conception to early childhood, i.e. prenatal development to child growth—when organogenesis and rapid growth are occurring^9^—is critical to the immediate and future health of the infant. Studies that looked at undernutrition acting in this early life period (as a result of either maternal undernutrition or protein/calorie restriction),^4^ showed that it not only retarded growth,^10^ but also induced lifelong changes in hormonal concentrations, and the sensitivity of various tissues to these fetal and placental hormones—alterations that lead to abnormal organ development^11,12^ and to diseases such as type-2 diabetes mellitus (T2DM), cardiovascular disease (CVD), kidney disease, obesity, hypertension, osteoporosis and metabolic syndrome in later life.^13,14^ These irreversible changes to tissue structure and physiology made to survive the harsh environment encountered in utero have also been called ‘programming’, and they are dependent on the nature and point at which exposure to the insult occurs, since tissues mature at different rates and time points.^11,15^ This differential effect is well illustrated with undernutrition, to which exposure too soon after conception, for example, slows down fetal growth and leads to low birthweight of the infant. In contrast, if undernutrition occurs during mid-pregnancy, it may alter placental development and lead to fetal wasting during the remainder of the pregnancy—disturbances that can result in distinct metabolic phenotypes in adulthood.^13^ Exposure to various other environmental factors including maternal stress, infections, hypertension, obesity, teratogens, alcohol, drugs, cigarette smoke, over nutrition and paternal malnutrition, within these critical windows of growth and development, have also been associated with an increased risk of adult disease.^16,17^

## Underlying mechanisms

The mechanisms that mediate the programming effects of diverse environmental insults, or how this memory is stored are unclear, but a few have been postulated. These include the following.

**Figure 1.**
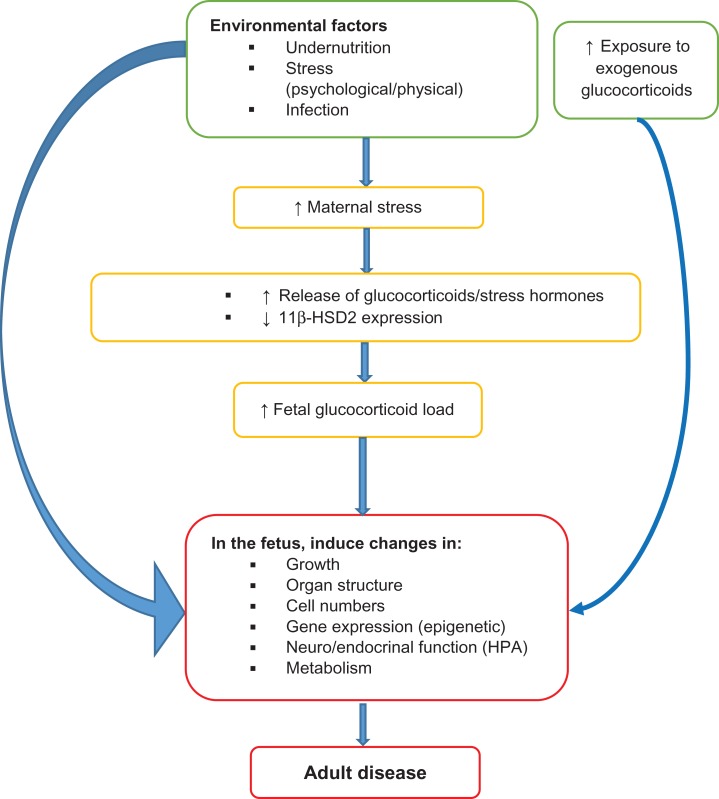
A theory of glucocorticoids as a potential common mechanism through which various environmental factors exert their programming effects.

1. *Excessive exposure to glucocorticoids (GCs):* GCs (stress hormones) are well known for their role in homeostasis (control of blood pressure and glucose metabolism) in adult life, but are also essential for fetal maturation.^18^ However, fetal GC load is normally regulated by 11β-hydroxysteroid dehydrogenase type-2 (11β-HSD2), a placental enzyme that inactivates GCs. Exposing the fetus to high GC levels (by inhibiting 11β-HSD2 or bypassing it through administration of synthetic GC) in rodents leads to growth retardation, and increases risk of glucose intolerance and high blood pressure in adulthood.^18,19^ Interestingly, other insults (notably maternal undernutrition) provoke a stress response (increasing maternal GC levels) and attenuate 11β-HSD2 expression—potentially increasing fetal exposure to GCs. Thus, GCs may provide a common mechanism through which other insults exert their programming effects (illustrated in Figure 1 below).
2. *Dysregulation in the development of the hypothalamic–pituitary–adrenal (HPA) axis:* A number of environmental exposures during early life (such as maternal stress, infections, undernutrition or GC treatment) have been shown to permanently increase activity of the HPA axis. This may result from differential expression of the GC receptors, with reduced expression in the hypothalamus attenuating negative feedback and increasing GC production from the adrenal glands. Changes in the activity of this neuroendocrine system influence development and regulation of various organs, and homeostatic systems, such as the central nervous, cardiovascular and metabolic systems, pancreas, kidney and adipose tissue. This aberrant activity can ultimately lead to increased risk of cardiovascular disease and cardio-metabolic disorders in adulthood
3. *Irreversible changes in organ structure:* Early life insults might also lead to permanent dysfunction and disease through irreversible changes in organ structure. For example, several insults such as undernutrition and hypoxia^20^ in utero have been associated with reduced nephron numbers (as a result of decreased nephrogenesis or renal progenitor cells),^21^ fewer pancreatic β-cell numbers/islet vascularization^22^ and liver lobules. The reduction in nephron numbers/function would increase risk of hypertension and renal disease, while reduced β-cell mass (as well as defective insulin signaling in skeletal muscle and adipose tissue),^23^ would dispose the individual to impaired glucose control in older age.
4. *Alterations in gene expression:* A more recently suggested mechanism through which early exposures might mediate their long-term effects, involves epigenetics.^3^ Epigenetic modifications, which include DNA methylation, histone marks and non-coding RNAs, regulate gene expression independent of changes in the DNA sequence, and are important to normal development and differentiation.^23^ DNA methylation, for example, is vital to cell differentiation, carcinogenesis and genomic imprinting.^24^ A number of studies in animal models and humans have shown that prenatal insults such as undernutrition or GCs can influence epigenetic marks. For example, in utero exposure to famine during the Dutch Hunger Winter of 1944–45, showed that these individuals in adulthood,^4^ had reduced methylation at regulatory regions for the insulin-like growth factor II, a hormone critical to growth and development.^25^ Other studies also found undernutrition in utero to alter methylation rates of the key enzyme (11β-HSD2) and hormonal receptor sites (GC receptors), changes that perturb hormone homeostasis and can lead to adult disease.^26^ Changes in non-coding RNA and histone modifications at genes of key transcription factors, such as the *PPARs*, *Hnf4α* and *Pdx1*, critical to normal tissue and organ development (adipose tissue, the pancreas, liver), cellular differentiation and metabolism, have also been associated with aberrance and susceptibility to T2DM.^27^

Other proposed mechanisms include genetics,^28^ cellular aging^29^ and intergenerational effects (exposures experienced by one generation that influence the health of the next, because they persist across generations or are genetic, or occur in utero and are self-perpetuating such as those that affect the HPA axis).^30^

## The impact on risk of NCDs: could DOHaD have a role?

It is known now that NCDs can be caused by a number of factors including genetics, environmental, physiological and behavioral patterns, and that they can occur in all age groups even though they are primarily diseases of old age.^31^ NCDs are the current leading cause of death globally, accounting for about 40 million (70%) deaths annually.^31^ They are generally attributed to four main risk factors: tobacco smoke, harmful alcohol consumption, poor diet and physical inactivity.^31^ However, these factors do not seem to fully explain the pattern of NCDs emerging in developing countries with the fast pace of urbanization, and consequent epidemiological and nutrition transitions.^32,33^ The epidemics in these regions seem to differ in some characteristics—with presentation occurring at a seemingly earlier age and disease progression at a faster rate than has been reported in developed countries;^34,35^ more than four-fifths of the estimated 15 million premature worldwide deaths from NCDs occur in these low- and middle income settings.^31^ These differences raise the question as to whether there are other drivers of chronic disease in these regions and the argument that there may well be.^36^ A proportion of NCDs in less developed resource-constrained countries could probably be explained by other factors, particularly,^4^ the encounter of adverse experiences during critical periods of growth (prenatal, childhood and in adolescence).^32^

Evidence from the numerous studies cited above would strongly support this notion, highlighting the need to further understand the role DOHaD may have in driving the NCD epidemic, and how it could contribute to the design of appropriate interventions, to address this growing public health problem,^32^ especially given that it is in these same regions that poverty, malnutrition, infections, low birth weight and poor sanitation are still prevalent. DOHaD science would be particularly useful for informing ways to improve nutrition and not just in early life, where fetal malnutrition, largely a consequence of poor maternal nutrition, has been shown to alter normal patterns of growth and development,^37^ but throughout the life course.^38^ Presently, about one-third of the world’s population, mostly in developing countries, suffers from some form of malnutrition; 815 million from calorie deficiency and nearly 2 billion from being overweight or obese.^39^ This year alone, maternal and child undernutrition accounted for 10% of the global burden of disease^40^ and obesity for about 2.8 million deaths.^41^ Together with other, often related NCDs, these represent a significant burden of ill health and put enormous strain not just on individuals and their families, but also on the health systems, societies and economies of these nations.^42^ Knowing this, it is imperative to step up the momentum to tackle these problems where effort is already being made, an even more importantly, to garner attention, as well as begin to utilize what knowledge of DOHaD we have, in regions where little is understood or being done.^34^

In conclusion, we now know that it is possible to reduce the burden of NCDs and to have an impact on long-term health outcomes by using approaches that address the influence of environmental factors on growth and development. Because these, unlike genetics or aging (important causes of NCDs), can be modified, they provide an opportunity, using the knowledge there is of developmental plasticity, to design interventions that could prevent many of these chronic diseases.^16^ However, like all previous successes, HIV-AIDs the classic one, progress addressing NCDs will require the political will, and NCDs getting on the national agendas, especially those of countries in the developing regions.

As for the next steps and interventions, it is critical that these take a life-course as well as a multi-disciplinary approach to be able to affect multiple generations.^31,34,43,44^ A few to consider include:

- *Reducing poverty*: which would improve living standards and health outcomes because it would reduce the risk of malnutrition,^3,44^ infections^32^ and disease.
- *Investing in nutrition*: to improve access, availability and affordability of nutritious foods.^45^
- *Education*: on the importance of nutrition and physical activity—using mass media for the general public and by integrating the subjects into science syllabi for primary and secondary school.
- *Promoting breast feeding, and healthy infant and young child feeding practices*.^44^
- *Improving practices/access to water, sanitation and hygiene*.
- *Supporting strategies to reduce tobacco smoking and alcohol consumption*.^42^
- *Addressing gender inequity* so that females are empowered to make better decisions for themselves and their families, the children particularly.^44^
